# Thumb Reconstruction Using Foucher’s Flap

**DOI:** 10.3889/oamjms.2016.017

**Published:** 2016-01-16

**Authors:** Nardi Kola

**Affiliations:** Service of Burns and Plastic Surgery, UHC Mother Teresa, Tirana, Albania

**Keywords:** Foucher’s flap, thumb, First DMCA, CMC joints, interosseus

## Abstract

**BACKGROUND:**

Extensive pulp defects of the thumb, with the exposure of tendon or bone, are challenging reconstructive problems. Surgical treatment includes the use of local, regional, and free flaps.

**AIM:**

This paper is focused in Foucher’s neuro vascular flap. First DMCA or Foucher’s pedicle flap is a successful thumb reconstruction method, especially in patients not disturbed by its cosmetic appearance.

**MATERIAL AND METHODS:**

The first dorsal metacarpal artery (FDMCA) arises from the radial artery in the first intermetacarpal space, just distal to the tendon of the extensor pollicis longus. Pulp area of the thumb is the area where Foucher’s flap is more utilizable. This technique has other applications such as first web reconstruction, thumb lengthening, and following resection of tumors on the dorsum of the hand.

**RESULTS:**

We have in study 7 cases with work related trauma in two years period of time, between 2012 and 2014. We had only one partial flap survival and all the other flaps survived entirely. We have also taken in consideration subjective satisfaction with a range score from 4 to 10, cold intolerance, flap area and donor site sensibility with a range score from low to medium to normal.

**CONCLUSION:**

Careful pedicle discovery, secured elevation, pedicle strangulation prevention are very important for flap survival.

## Introduction

Extensive pulp defects of the thumb, with the exposure of tendon or bone, are challenging reconstructive problems. Surgical treatment includes the use of local, regional, and free flaps.

Moberg flap, Littler neurovascular flap, Kutler’s V-Y flaps, Foucher’s flap, and free flaps including toe to thumb transfer are best surgical choices.

This paper is focused in Foucher’s neuro vascular flap. The innerved first dorsal metacarpal artery flap from the dorsum of the index finger was first described by Hilgenfeldt. An island flap carried on a neurovascular pedicle consisting of the first dorsal metacarpal artery was first demonstrated by Foucher [1]. Despite a successful reconstruction, the thumb may never return to a pre-injury level of function. The patient and surgeon should be aware of this possibility.

First DMCA or Foucher’s pedicle flap is a successful thumb reconstruction method, especially in patients not disturbed by its cosmetic appearance.

The first dorsal metacarpal artery (FDMCA) arises from the radial artery in the first intermetacarpal space, just distal to the tendon of the extensor pollicis longus. The artery divides into the radial branch to the thumb, the intermediate branch to the first web space, and the ulnar branch to the index finger. In 90% of cases, the FDMCA parallels the second metacarpal bone distally. In 10% of cases, it is found in the midline of the triangle that is formed by the first commissura. The FDMCA is in a superfascial (57%) or subfascial (43%) location [1-3]. The outer diameter of the artery at its widest point is 1.0 ± 1.5 mm. In those without a palmar branch the diameter is less than 1 mm. A muscular artery is present in 40%. Flap pedicle is about 5-9 cm. The length of the flap varies from 2-4 cm.

## Material and Methods

We have in study 7 cases with work related trauma in two years period of time, between 2012-2014. Two of the cases were presented in our emergency with partial distal thumb amputation and five other this distal open wounds with bone exposure.

The patients mean age was 31 (range 17-48 years), and mean follow-up was 11 months (range 6-17). All the patients were males. Emergency surgery was performed in all patients with a time delay after injury of 11-25 hours. The minimum defect was 14 x 19 mm and the maximum 20 x 38 mm. Pedicle length was 51-93 mm. We had only one partial flap survival and all the other flaps survived entirely. We have also taken in consideration subjective satisfaction with a range score from 4 to 10, cold intolerance, flap area and donor site sensibility with a range score from low to medium to normal.

## Results

Selecting the most appropriate technique for thumb reconstruction depends on multiple factors, including the following: Level of injury; Status of the remaining hand; Presence or absence of the thenar musculature; Age, occupation, overall health, and functional demands of the patient.

Pulp area of the thumb is the area where Foucher’s flap is more utilizable. In cases of traumatic injury to the thumb, radiographs should be obtained to determine the presence of fracture and to assess the quality of the IP, MCP, and CMC joints.

The patient is placed in a supine position, and the arm is positioned on the arm table. The operation is done in plexus block or under general anesthesia. A tourniquet (250–300 mmHg) is applied at the upper arm. After debridement of the thumb defect, the skin flap is outlined. The flap is harvested from the dorsal aspect of the index finger, including the first dorsal metacarpal artery and a branch of the superficial radial nerve as a pedicled flap. First DMCA is identified at the emerge point between the two heads of the first dorsal interosseous muscle in te index finger.

The fascia is cut and the periosteum is then stripped off the second metacarpal bone on the radial side. The nutrient branch to the metacarpal head is identified and tied up. The flap is elevated, leaving the paratenon intact. The pedicle includes the fascia of the first dorsal interosseus muscle, the dorsal veins, and the sensory branch of the radial nerve. Then, the flap is placed over the defect taking. The donor area is covered by an antecubital full-thickness skin graft. A lot of attention should be taken to the pedicle positioning in order to prevent strangulation [4-6].

The predicted outcome of surgery generally favors reconstruction when an amputation has occurred distal to the MCP joint and has therefore left the first web space, as well as the thenar muscles (including their insertions), preserved.

This technique has other applications such as first web reconstruction, thumb lengthening, and following resection of tumors on the dorsum of the hand [5, 7].

Individual results of the seven patients with thumb reconstruction using Foucher’s flap are shown in Table 1.

**Table 1:** Individual results of the seven patients with thumb reconstruction using Foucher’s flap

Patient	1	2	3	4	5	6	7
Sex	Male	Male	Male	Male	Male	Male	Male

Age	25	28	48	17	36	43	39

Surgery time after truauma	11 hours	16 hours	21 hours	25 hours	18 hours	22 hours	15 hour

Flap survival	Total	Total	Partial	Total	Total	Total	Total

Pedicle measured	68mm	65mm	51 mm	72mm	80mm	93 mm	85mm

Cold intolerance	No	No	Jes	No	No	No	No

Satisfaction	10	9	8	9	10	10	9

Flap sensibility	Normal	Normal	Medium	Normal	Normal	Normal	Medium

Donor site sensibility	Medium	Normal	Medium	Normal	Normal	Normal	Medium

**Figure 1:**
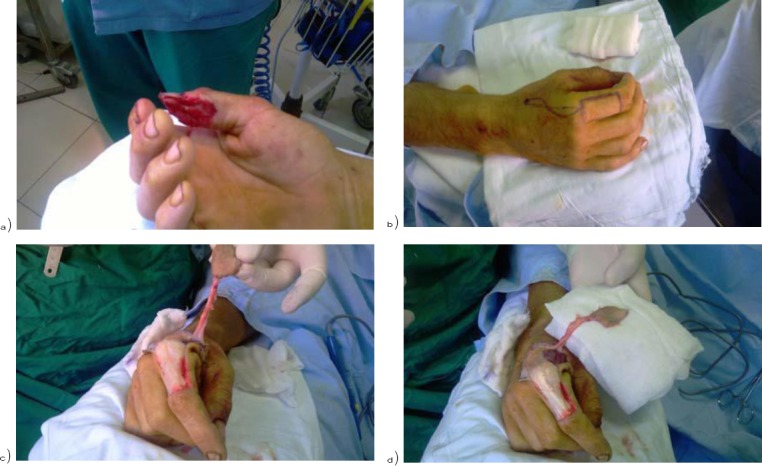
*a) and b) Case display [a 25 years old boy with a work-related injury]; c) and d) flap elevation*.

**Figure 2:**
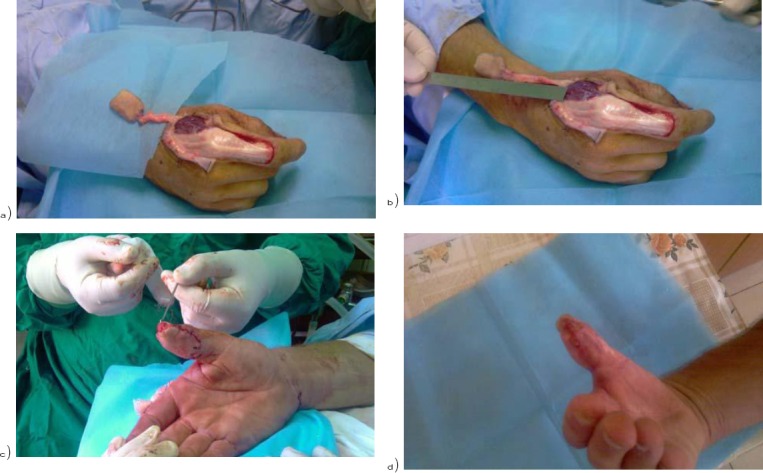
*a) and b) pedicle length is measured; c) wound closure; d)after three weeks*.

Details of the patient with thumb reconstruction using Foucher’s flap are shown in Fig. 1 and Fig. 2 [a 25 years old boy with a work-related injury].

In conclusion, Foucher’s flap is a reliable choice in thumb reconstruction surgery. The results are very good and complications are rarely seen. Careful pedicle discovery, secured elevation, pedicle strangulation prevention are very important for flap survival.
